# The integrated stress response in pancreatic β-cell failure in diabetes: from molecular mechanisms to precision therapeutics

**DOI:** 10.3389/fendo.2026.1855305

**Published:** 2026-08-19

**Authors:** Yijun Huang, Yu Zeng, Xinying Zhong, Lin Li

**Affiliations:** 1 The First Clinical College of Zunyi Medical University, Zunyi, China; 2 Department of Cell Biology, School of Preclinical Medicine, Zunyi Medical University, Zunyi, China

**Keywords:** diabetes mellitus, eIF2α phosphorylation, endoplasmic reticulum stress, integrated stress response (ISR), pancreatic β-cells

## Abstract

Diabetes mellitus is a global metabolic disorder characterized by progressive pancreatic β-cell failure, which leads to inadequate insulin secretion, often in combination with insulin resistance, and ultimately disrupts glucose homeostasis. The Integrated Stress Response (ISR), a crucial signaling network that enables cells to maintain homeostasis under endogenous and exogenous pressures, has garnered increasing attention for its role in regulating islet β-cell function and contributing to their failure. In recent years, a growing body of evidence has revealed that the ISR acts as a dual-edged sword in the onset and progression of diabetes. On the one hand, ISR activation can transiently relieve biosynthetic stress and help preserve cellular homeostasis. On the other hand, persistent or dysregulated ISR signaling is increasingly associated with impaired insulin production, loss of β-cell identity, and cell death in diabetes. In this Review, we summarize the molecular architecture of the ISR, focus on its regulatory role in pancreatic β-cell stress biology, and examine how ISR-related pathways are engaged in monogenic diabetes, type 1 diabetes, and type 2 diabetes. We further evaluate current therapeutic strategies targeting ISR-associated pathways and highlight the central translational challenge in this field, aiming to provide a theoretical basis and novel insights for a deeper understanding of the molecular mechanisms driving diabetic β-cell failure and for the development of novel precision therapeutic approaches.

## 1 Introduction

The global prevalence of diabetes mellitus (DM) represents one of the most formidable public health challenges of the 21st century. According to the latest data from the International Diabetes Federation, there were 589 million adults aged 20–79 years with diabetes globally in 2024, and projections indicate that by 2050, the total number of individuals with diabetes will rise to 853 million (1). Diabetes is characterized by chronic hyperglycemia resulting from insulin resistance and/or β-cell failure (2). Among these, the progressive failure of β-cell function is a common feature of both type 1 and type 2 diabetes (3). The mechanisms underlying this failure are complex and have not yet been fully elucidated, making it one of the central challenges in diabetes research.

Recent advances in research have revealed that the Integrated Stress Response (ISR), an evolutionarily highly conserved cellular defense mechanism (4), is critically involved in regulating the function and fate of pancreatic islet β-cells. The ISR senses multiple intracellular stress signals, such as endoplasmic reticulum stress, oxidative stress, and inflammatory signals, and adjusts gene expression and protein synthesis through a series of signal transduction pathways to maintain intracellular homeostasis (5, 6). However, when stress becomes chronic or its regulation is disrupted, ISR signaling can shift towards a pro-apoptotic and dedifferentiation mode (7). In pancreatic β-cells, this chronic activation of the ISR is currently being investigated and is suggested as a potential significant contributor to the development of diabetes.

Therefore, in-depth analysis of the central role and molecular mechanisms of the ISR in diabetes-associated islet β-cell failure not only helps to reveal new mechanisms underlying the onset and progression of diabetes but also provides an important theoretical basis and potential direction for the development of precision therapeutic strategies targeting the ISR. In this Review, we summarize the molecular framework of the ISR, discuss its role in pancreatic β-cell stress responses, and examine its involvement across different forms of diabetes. We also address the therapeutic potential and challenges of targeting ISR-associated pathways for the preservation of β-cell function.

## 2 ISR molecular network

Current research indicates that the Integrated Stress Response (ISR) is a highly conserved adaptive response mechanism activated by cells in response to various internal and external stress stimuli (8). Its core lies in integrating signals from multiple stress-sensing kinases through the phosphorylation of eIF2α, thereby coordinating the regulation of gene expression at both the translational and transcriptional levels to maintain intracellular homeostasis or induce cells to enter specific functional states (9).

### 2.1 Components of ISR

**Figure 1:**
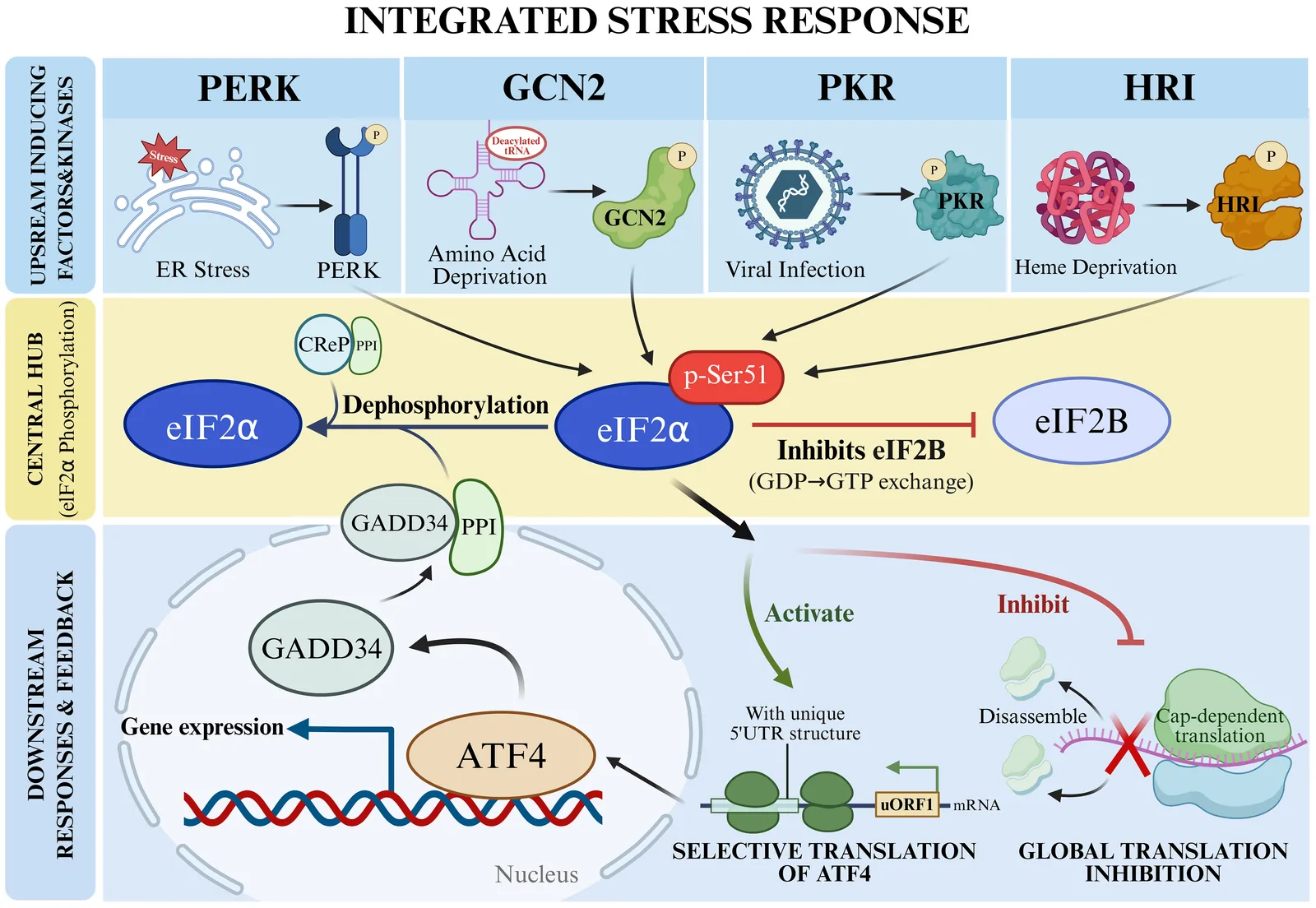
Molecular framework of the integrated stress response (ISR) PERK, GCN2, PKR, and HRI areactivated by distinct stress stimuli and converge on eIF2α Ser51 phosphorylation, the centralhub of ISR signaling. Phosphorylated eIF2α suppresses eIF2B activity, thereby reducing globalprotein synthesis while selectively enhancing ATF4 translation. ATF4 then drives downstreamtranscriptional responses that coordinate stress adaptation and cell fate decisions. ISR signalingis resolved through GADD34/CReP–PP1-dependent eIF2α dephosphorylation, which restorestranslational equilibrium. Arrows indicate activation, and blunt-ended lines indicate inhibition.Created in BioRender. Huang, Y. (2026) https://BioRender.com/3tfn318.

The core components of the ISR include stress-sensing kinases, the key signal transduction molecule eIF2α, and downstream transcriptional regulators. Specifically, four main members of the stress-sensing kinase family have been identified to date: PKR-like ER kinase (PERK), double-stranded RNA-dependent protein kinase (PKR), heme-regulated eIF2α kinase (HRI), and general control non-derepressible 2 (GCN2) (**Figure 1**) (10). The reversible phosphorylation of eukaryotic initiation factor 2 alpha (eIF2α) is the central event of the ISR (11). Upon activation by any of these stress-sensing kinases, eIF2α is phosphorylated at Ser51, which arrests the formation of the translation initiation complex but selectively promotes the synthesis of adaptive transcription factors such as ATF4 (12). This triggers a response characterized by “global translational inhibition coupled with specific translational activation,” thereby ensuring that the ISR can mount a precise and coordinated reaction to various changes in the internal and external environment (9, 13, 14).

### 2.2 ISR activation

ISR activation is a conserved adaptive response through which diverse internal and external stressors are integrated into a common signaling output that centers on eIF2α phosphorylation (15). Despite the diversity of these upstream stress signals, ISR initiation in mammalian cells is mediated by four eIF2α kinases - PERK, PKR, HRI and GCN2 - each of which responds to distinct cues but converges on the same downstream pathway (16).

PERK, located on the endoplasmic reticulum (ER) membrane, is the primary sensor of ER stress (17). It is activated when unfolded or misfolded proteins accumulate in the ER lumen or when metabolic, calcium, or redox homeostasis is disrupted. PERK activation has classically been associated with its dissociation from the ER chaperone BiP (binding immunoglobulin protein) (18).

The classic trigger for GCN2 activation is amino acid deprivation. When the intracellular amino acid level is insufficient, deacylated tRNA will bind to and activate GCN2, which in turn phosphorylates eIF2α (19). However, recent studies have challenged this view, suggesting that GCN2 activation is not restricted solely to amino acid deficiency, and placing ribosomes at the very center of the process by which GCN2 mediates the activation of the ISR (20).

PKR is primarily activated by viral double-stranded RNA (dsRNA), leading to the inhibition of viral mRNA translation and the promotion of apoptosis in response to viral infection. However, PKR activation is not restricted to viral infections; it can also be triggered by other stress signals, such as oxidative stress and ER stress (14).

HRI was initially reported to be activated upon heme deficiency (14). Nevertheless, further research indicates that cytosolic proteotoxicity, resulting from oxidative stress, osmotic stress, heat shock, or proteasome inhibition, constitutes the primary trigger for HRI-induced ISR (21).

### 2.3 Downstream translational and transcriptional responses

Although the four kinases respond to distinct stress signals, they ultimately converge on the common node of eIF2α phosphorylation (9). Phosphorylation of eIF2α inhibits the eIF2B-mediated exchange of eIF2-GDP to eIF2-GTP, thereby reducing the formation of the ternary complex composed of eIF2-GTP and methionyl-initiator tRNA (22). This leads to a reduction in global protein synthesis. However, the effect of phosphorylated eIF2α (p-eIF2α) is not merely one of global inhibition; it simultaneously and selectively activates the translation of key transcription factors, such as ATF4 (23). The mRNA of ATF4 contains a unique 5’ untranslated region (5’UTR) structure that enables its translation efficiency to be enhanced under conditions of eIF2α phosphorylation (24). ATF4, as a crucial transcription factor, subsequently regulates the expression of a series of downstream target genes. These targets include molecular chaperones (e.g., BiP) that contribute to cell survival and stress adaptation, as well as the pro-apoptotic factor C/EBP homologous protein (CHOP) (25). This sophisticated mechanism of translational reprogramming allows cells, when responding to stress, to simultaneously reduce the synthesis of unnecessary proteins while prioritizing the production of key proteins required to cope with the stress, in an attempt to restore cellular homeostasis. However, when stress is prolonged or excessive, the upregulation of CHOP expression drives cells toward apoptosis (26). Furthermore, the termination of the ISR depends on the dephosphorylation of p-eIF2α, a process in which protein phosphatase 1 (PP1) and its regulatory subunits, GADD34 and CReP, constitute a critical negative feedback axis (9). During the later stages of the stress response, ATF4 and CHOP can induce the upregulation of GADD34 expression. Subsequently, GADD34 forms a complex with PP1, promoting the dephosphorylation of p-eIF2α and thereby restoring protein synthesis (27). In contrast to stress-induced GADD34, CReP is continuously expressed under non-stressed conditions and primarily functions to maintain low basal levels of p-eIF2α, thus sustaining translational homeostasis (28).

## 3 Mechanistic regulation of ISR in pancreatic β-cells

### 3.1 The unique basis of β-cell ISR sensitivity

The exceptional sensitivity of pancreatic β-cells to the Integrated Stress Response (ISR) is not coincidental but rather stems from their unique structural and metabolic characteristics. These intrinsic biological properties render β-cells more susceptible to pathological ISR activation upon exposure to various stresses, thereby accelerating their functional decline and cell death. To maintain precise and continuous blood glucose regulation, pancreatic β-cells must sustain an extremely high load of proinsulin synthesis and secretion over the long term. To meet this immense biosynthetic demand, each β-cell can synthesize up to 6,000 preproinsulin molecules per second, making proinsulin biosynthesis a substantial metabolic burden that accounts for 30% to 50% of total protein synthesis in β-cells (29). This functional demand drives the formation of an extensive endoplasmic reticulum (ER) network, placing the β-cell ER in a state of constant high demand and rendering it more prone to protein folding disturbances (29, 30). Under conditions of high glucose stimulation or increased metabolic stress, the rate of protein synthesis in β-cells can rapidly increase. When this rate exceeds the folding and processing capacity of the ER, unfolded or misfolded proteins accumulate quickly, thereby activating the PERK–eIF2α pathway and triggering the Integrated Stress Response (ISR) to transiently inhibit translation and restore proteostasis (31, 32). These features explain why ISR activation is not an incidental event in β-cells, but a predictable consequence of their highly specialized secretory physiology.

### 3.2 Adaptive activation of ISR: a protective mechanism of pancreatic β-cells

During endoplasmic reticulum (ER) stress, pancreatic β-cells mobilize the unfolded protein response (UPR), thereby activating the Integrated Stress Response (ISR) to adapt to changes in metabolic and secretory demands by transiently inhibiting protein translation and reducing the protein synthesis load (32, 33). It should be noted that the UPR and the ISR are closely related but not identical concepts.

The UPR is mediated by three ER transmembrane sensors: inositol-requiring enzyme 1 (IRE1), protein kinase R-like endoplasmic reticulum kinase (PERK), and activating transcription factor 6 (ATF6) (34). In contrast, the ISR is a broader translational control program that converges on eIF2α phosphorylation in response to multiple forms of cellular stress (35, 36). PERK represents a major point of overlap between these two pathways, which explains why ER stress and ISR activation are tightly linked (37). During ER stress, PERK-mediated eIF2α phosphorylation transiently attenuates global protein synthesis while permitting the selective translation of ATF4 and the induction of adaptive stress-response genes (38, 39). Experimental blockade of eIF2α phosphorylation (e.g., via the eIF2α S51A mutation or PERK deletion) leads to excessive protein synthesis and misfolding, increased oxidative stress, and impaired β-cell function, suggesting that short-term or moderate ISR activation helps alleviate ER load and maintain secretory homeostasis (6, 40). Experimental studies in mouse islets and insulin-secreting cells found that acute glucose stimulation was accompanied by a PP1-dependent reduction in net eIF2α Ser51 phosphorylation. This change was associated with increased global translation (41). However, these findings do not prove that glucose directly activates PP1 or that this mechanism applies to all β-cell states. Together, the results are consistent with a glucose-associated change in the balance between eIF2α phosphorylation and dephosphorylation. Such reversible regulation may help coordinate protein synthesis with ER folding capacity under acute nutrient stimulation. However, under chronic metabolic stress, this adaptive coordination may be progressively disrupted, contributing to sustained ISR activation and β-cell dysfunction.

### 3.3 Mechanism of ISR amplification in diabetic pancreatic β-cells and its cytopathological consequences

In the diabetic metabolic environment, organelles within pancreatic β-cells form an interconnected network through membrane contact sites and signaling molecules. This inter-organelle communication initially supports cellular adaptation. However, under persistent stimulation by high glucose and lipotoxicity (glucolipotoxicity), it can amplify unresolved stress signals and promote sustained UPR/ISR activation. This transition progressively impairs β-cell function and survival.

#### 3.3.1 Feedback amplification of endoplasmic reticulum stress by mitochondrial damage

**Figure 2:**
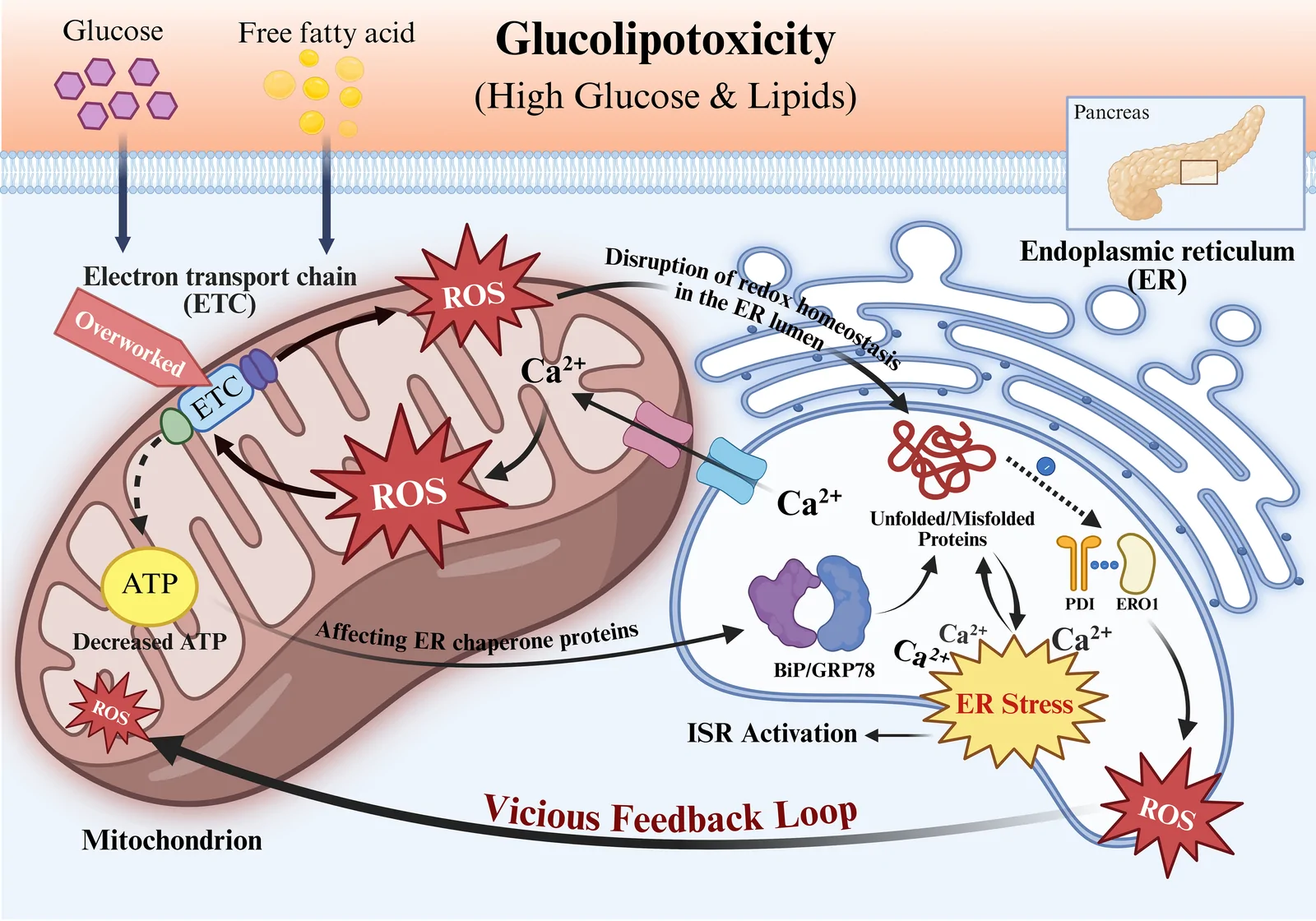
Feedback amplification of endoplasmic reticulum stress by mitochondrial damage Chronic exposureto high glucose and free fatty acids overloads mitochondrial oxidative metabolism, leading toexcessive ROS generation and reduced ATP availability. Mitochondrial oxidative stress and energydeficiency disrupt ER redox homeostasis and compromise ATP-dependent chaperone-assisted protein folding, thereby promoting the accumulation of unfolded or misfolded proteins and inducing ER stress. In parallel, Ca^2+^ transfer from the ER to mitochondria further enhances mitochondrial ROS production, whereas oxidative protein folding within the ER, mediated by PDI and ERO1, generates additional ROS. Together, these processes form a self-amplifying ER–mitochondria stress circuit that perpetuates ISR activation and contributes to β-cell proteostatic failure and functional deterioration. Arrows indicate activation or propagation. Created in BioRender. Huang, Y. (2026) https://BioRender.com/po1s2vk.

In the glucolipotoxic microenvironment of diabetes, mitochondria are major targets of metabolic stress and can also exacerbate endoplasmic reticulum (ER) stress (**Figure 2**). The two form a vicious feedback loop through physical connections and biochemical signals. Under the persistent hyperglycemic conditions of diabetes, β-cells rely heavily on mitochondrial oxidative metabolism to drive glucose-stimulated insulin secretion (GSIS). This places the electron transport chain (ETC) under a continuous, high workload, thereby significantly increasing the production of mitochondria-derived reactive oxygen species (ROS) (42, 43). Excessive mitochondrial ROS not only cause oxidative damage to the mitochondria themselves but can also diffuse and disrupt the redox homeostasis within the ER lumen. The resulting impairment of protein disulfide-bond formation promotes the accumulation of unfolded or misfolded proteins and activates ER stress (44). Concurrently, impaired mitochondrial function reduces ATP production. Since ER chaperone proteins (such as BiP/GRP78) and their co-factors are highly dependent on ATP, this energy deficit limits the efficiency of ER protein folding, which would further intensify UPR activation (43, 45, 46).

Furthermore, mitochondria and the endoplasmic reticulum form close physical contacts through mitochondria-associated membranes (MAMs) (47). In β-cells, this structural coupling is crucial for coordinating cellular functions, as it facilitates the efficient transfer of calcium ions (Ca2+) between the ER and mitochondria (48). However, under ER stress conditions, excessive mitochondrial Ca^2+^ uptake stimulates the tricarboxylic acid cycle and oxidative phosphorylation. This further increases mitochondrial ROS production (42). Persistent ER stress also drives oxidative protein folding. During this process, protein disulfide isomerase (PDI) transfers electrons to the ER oxidoreductin 1 (ERO1), with molecular oxygen serving as the terminal electron acceptor. This reaction generates ER-derived ROS (49). Consequently, ER-derived ROS exacerbate the mitochondrial oxidative burden in return, causing mitochondrial dysfunction and ER stress to reinforce each other. A self-amplifying, vicious feedback loop is thus established between the two (49, 50). This interconnected causal network rapidly amplifies local mitochondrial damage through ER stress into cell-wide ISR activation, thereby accelerating the collapse of proteostasis and functional failure in pancreatic β-cells.

#### 3.3.2 Direct regulation of nuclear gene expression by endoplasmic reticulum stress

ER stress remodels nuclear gene expression through the PERK, IRE1α, and ATF6 branches of the UPR. These pathways induce molecular chaperones, ER-associated degradation (ERAD) components, and metabolic regulators that support cellular adaptation (51). Although this transcriptional response initially supports the restoration of proteostasis, chronic ER stress conditions associated with diabetes can lead to its persistent dysregulation and contribute to progressive β-cell dysfunction (52). Within this chronic response, sustained PERK–eIF2α–ATF4 signaling has been associated with reduced expression of genes involved in insulin synthesis and β-cell identity (53, 54). Although transient ATF4 activation supports stress adaptation, prolonged signaling under glucolipotoxic conditions can promote CHOP expression and shift the transcriptional program toward functional impairment and cellular injury (55). Concurrently, IRE1α, one of the most conserved sensors of the UPR, possesses dual kinase and endoribonuclease (RNase) activities (56). During transient endoplasmic reticulum (ER) stress, the RNase activity of IRE1α primarily functions by splicing XBP1 mRNA to generate the transcription factor XBP1s (spliced X-box binding protein 1). XBP1s expands the secretory and folding pathways, thereby accommodating the temporarily increased protein load. This is considered a protective UPR output (57). However, under the chronic metabolic conditions associated with diabetes, excessive ER stress can lead to sustained, high-intensity activation of the IRE1α pathway. This process inhibits XBP1s and induces the degradation of insulin mRNA, thereby impairing insulin synthesis capacity and exacerbating β-cell dysfunction (58). Together, persistent PERK–ATF4 and IRE1α signaling shift the nuclear response from short-term adaptation toward long-term dysfunction and apoptosis.

#### 3.3.3 The overall negative impact of sustained ISR activation on islet β-cell fate

When inter-organelle stress remains unresolved, the initially adaptive UPR/ISR becomes chronically activated. This transition promotes β-cell functional failure and programmed cell death (9, 59). A key effector of this pathological shift is the transcription factor CCAAT/enhancer-binding protein homologous protein (CHOP), whose sustained induction promotes pro-apoptotic gene expression (60). At the cellular level, CHOP activation has been shown to reduce the expression of the anti-apoptotic protein BCL-2. It also promotes the upregulation of several BH3-only proteins (e.g., BIM, PUMA) as well as the death receptor DR5. Together, these changes promote mitochondrial outer membrane permeabilization and death receptor-mediated apoptotic signaling (61). Consistent with these pro-apoptotic effects, genetic inhibition of CHOP in β-cells has been shown in animal models to alleviate ER stress-related islet dysfunction (62). Simultaneously, sustained UPR/ISR activation can also lead to decreased expression of key β-cell-specific transcription factors (such as PDX1 and MAFA). Mature β-cells may consequently enter a dedifferentiated state characterized by impaired insulin secretion and progenitor-like features. This loss of identity reduces functional β-cell mass and worsens glucose dysregulation (63). Furthermore, persistent ER stress can significantly induce the expression of TXNIP (Thioredoxin-interacting protein). TXNIP promotes the production of IL-1β by activating the NLRP3 inflammasome, thereby driving inflammatory responses within the islets and exacerbating β-cell dysfunction and death (64). Studies have indicated that the upregulation of TXNIP induced by ER stress is mediated by both the IRE1α and PERK-eIF2α pathways of the UPR in β-cells (65). Therefore, under diabetic conditions, sustained ISR collectively accelerates β-cell dysfunction and loss of identity through organelle stress amplification, apoptosis, dedifferentiation, and inflammation. These critical nodes (e.g., CHOP, TXNIP) offer potential directions for future intervention strategies.

In summary, it can be inferred that the ISR possesses a dual nature in β-cells. Transient activation reduces the secretory burden and supports proteostasis, whereas prolonged or dysregulated activation is associated with proinsulin misfolding, impaired insulin production, loss of β-cell identity, and apoptosis. However, significant knowledge gaps remain regarding the precise thresholds, timing, and reversibility of these processes, warranting more systematic investigation in the future.

## 4 The role of ISR in different types of diabetes

### 4.1 Monogenic diabetes: gene-driven ISR

**Figure 3:**
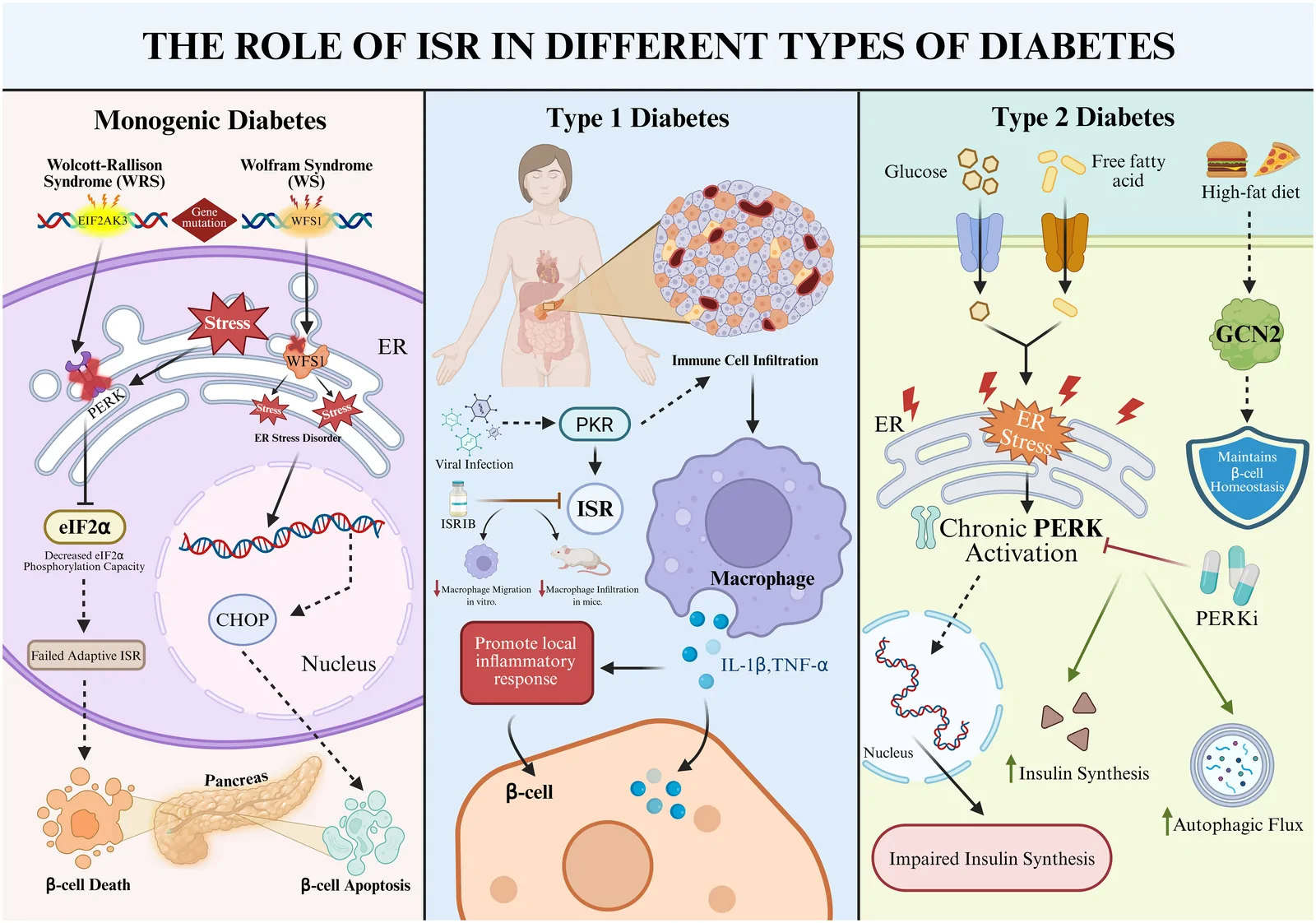
The role of ISR in different types of diabetes. In monogenic diabetes, mutations in EIF2AK3 orWFS1 directly disrupt ER homeostasis and ISR regulation, leading to β-cell dysfunction andapoptosis. In type 1 diabetes, inflammatory and antiviral cues activate ISR, particularly through PKR, thereby promoting macrophage activation, local inflammatory responses, and β-cell injury. In type 2 diabetes, chronic glucolipotoxic stress drives sustained PERK activation and maladaptive ISR signaling, resulting in impaired insulin synthesis and β-cell failure, whereas GCN2 may help preserve β-cell homeostasis under nutrient excess. Together, these findings highlight the context-dependent nature of ISR in diabetes, with adaptive or pathogenic effects determined by disease setting and stress duration. Arrows indicate activation or promotion, and blunt-ended lines indicate inhibition. Created in BioRender. Huang, Y. (2026) https://BioRender.com/13ps7zy.

Monogenic forms of diabetes, particularly those directly linked to endoplasmic reticulum stress and ISR pathways, provide valuable genetic evidence for understanding the crucial role of the ISR in β-cells (**Figure 3**).

1. Wolcott-Rallison Syndrome (WRS): Wolcott-Rallison Syndrome is a rare autosomal recessive disorder caused by mutations in the EIF2AK3 gene encoding the PERK kinase. Patients present with non-autoimmune insulin-dependent diabetes mellitus in the neonatal or early infant period (66). Under conditions of ER stress, the loss of PERK function and the consequent impairment of eIF2α phosphorylation in WRS patients lead to β-cell death, resulting in severe insulin deficiency and diabetes. This underscores that the PERK-mediated adaptive UPR/ISR is indispensable for the maintenance of pancreatic β-cell function (66, 67). Studies in mouse and cellular models have shown that Perk deletion or functional deficiency leads to islet ER dysfunction, abnormal insulin folding, and impaired β-cell function, further supporting the molecular basis of the human WRS phenotype (68, 69).
2. Wolfram Syndrome (WS): Wolfram Syndrome is a more complex autosomal recessive disorder caused by mutations in the WFS1 gene, with clinical manifestations including diabetes mellitus, optic atrophy, and diabetes insipidus (70). The WFS1 protein, encoded by this gene, is localized to the endoplasmic reticulum membrane and participates in maintaining ER calcium homeostasis and regulating the ER stress response (71). Its absence leads to dysregulated ER stress and activation of CHOP-mediated apoptotic pathways, which is associated with progressive β-cell failure (72). Recent studies utilizing stem cell-derived islets (SC-islets) and β-cell-specific *Wfs1* knockout mouse models have demonstrated that an activated Integrated Stress Response (ISR) is a key mechanism underlying β-cell failure in WS. This is characterized by enhanced eIF2α phosphorylation and PERK–ATF4 signaling, accompanied by decreased insulin content and impaired glucose-stimulated insulin secretion (GSIS). Importantly, this phenotype could be partially reversed by the ISR inhibitor ISRIB (73). This research provides crucial proof-of-concept for therapeutic strategies targeting the ISR, particularly highlighting the potential application of ISRIB.

### 4.2 Type 1 diabetes: immune-driven ISR

Type 1 diabetes (T1D) is an autoimmune disease characterized by the interplay of innate and adaptive immune responses within the pancreatic islets, leading to β-cell destruction (74). A hallmark pathological feature is immune cell infiltration (insulitis), with macrophages being among the early infiltrating cells in the islets. These inflammatory macrophages secrete IL-1β, TNF-α, and reactive oxygen species, and participate in T cell activation through MHC-II-mediated antigen presentation, thereby promoting local inflammation and the autoimmune process (75–77). Recent studies have revealed that the Integrated Stress Response (ISR) is activated in immune cells during T1D: PKR (an upstream ISR kinase), an eIF2α kinase induced by viral double-stranded RNA and interferons, shows significantly elevated expression in the islets of T1D patients (74). Furthermore, treatment with ISRIB reduces the migration of inflammatory macrophages *in vitro* and decreases macrophage infiltration into the islets of pre-diabetic female non-obese diabetic (NOD) mice (78). Inhibiting PKR recapitulates the effects of ISRIB, whereas blocking PERK (another upstream ISR kinase) has little impact, suggesting that PKR-driven ISR may play a significant role in the macrophage-mediated inflammatory response in T1D (78). Inhibition of the ISR (using ISRIB) also resulted in increased PD-L1 expression on macrophages and elevated PD-L1 levels within the insulitic lesions of NOD mice (78). This suggests that the ISR is not only involved in amplifying inflammation but may also influence the immune pressure on β-cells by modulating immune checkpoints.

### 4.3 Type 2 diabetes: metabolite-driven ISR dysregulation

Type 2 diabetes (T2D) is a heterogeneous disease characterized by varying degrees of β-cell dysfunction, often accompanied by insulin resistance (79). A common feature in T2D is the presence of endoplasmic reticulum stress and aberrant activation of the unfolded protein response (UPR) in β-cells, a process that can be triggered by factors such as free fatty acids, high glucose, islet amyloid polypeptide (IAPP) aggregation, and increased secretory demand due to insulin resistance (5). Among these, the high-glucose and high-lipid environment, termed “glucolipotoxicity,” is a major driver of β-cell dysfunction in type 2 diabetes (80). Studies in human islets have revealed that chronic exposure to high glucose and free fatty acids significantly activates the PERK signaling pathway, leading to impaired insulin synthesis (81). Notably, this chronic, high-level PERK activation is not protective but rather constitutes a pathogenic mechanism: treating islets under glucolipotoxic conditions with a PERK inhibitor (PERKi) not only partially restored insulin secretion but also increased autophagic flux (manifested as increased LC3 conversion and downregulation of P62 levels, which were inhibited by glucolipotoxicity) (81). Beyond PERK, another upstream ISR kinase, the amino acid-sensing kinase GCN2 (EIF2AK4), also plays a significant role in nutrient sensing. Research indicates that a high-fat diet increases phosphorylated GCN2 levels in mouse islets, and GCN2-deficient mice on a high-fat diet exhibit glucose intolerance and a reduction in islet β-cell mass, suggesting a protective role for GCN2 in β-cell homeostasis under conditions of nutrient overload (82).

In summary, genetic defects, immune inflammation, and metabolic overload can individually or synergistically act on different eIF2α kinase nodes, leading to ISR dysregulation. This, in turn, causes β-cell folding/secretory defects, inflammation amplification, and cell loss. The ISR exhibits both short-term protective and potential long-term pathogenic roles across different pathological contexts, suggesting that the key to restoring β-cell homeostasis lies in the precise modulation of the ISR pathway rather than its simple blockade. Based on this premise, the following section will explore how different intervention strategies targeting key nodes of the ISR can be developed, aiming to facilitate the translation from mechanistic understanding to clinical therapy.

## 5 Therapeutic strategies targeting ISR: translational prospects from bench to bedside

The Integrated Stress Response (ISR) plays a crucial role in regulating endoplasmic reticulum homeostasis, protein translation, and cell survival in β-cells. Its dysregulation is closely associated with β-cell dysfunction and the pathogenesis of diabetes, positioning it as a potential therapeutic target. However, its effects are context dependent. Transient activation can reduce the secretory burden and support cellular adaptation, whereas prolonged or dysregulated signaling contributes to β-cell dysfunction. Therapeutic strategies should therefore attenuate maladaptive ISR signaling without abolishing its basal adaptive functions. Current approaches include partial modulation of upstream kinases, regulation of downstream translational control, reduction of ER folding stress, and repurposing of drugs that improve the cellular stress environment.

### 5.1 Modulating PERK signaling: therapeutic potential and safety challenges

PERK is a key kinase in the ER stress-initiated ISR, playing a fundamental role in regulating proinsulin folding, translation rates, and β-cell homeostasis. Its dysregulation can lead to disrupted protein synthesis and impaired β-cell function (9, 83). Human genetic evidence also indicates that complete loss of PERK activity is detrimental. Mutations in the EIF2AK3 gene lead to PERK deficiency and, consequently, Wolcott-Rallison syndrome, a condition characterized by neonatal diabetes and pancreatic abnormalities (66, 84). This indicates that complete PERK blockade is unlikely to be a viable therapeutic strategy and provides a rationale for investigating partial modulation of excessive PERK signaling.

Currently, selective PERK inhibitors (e.g., GSK2606414) have been developed and utilized to study the functional regulation of PERK signaling (85). In metabolic stress models induced by high glucose and fatty acids, low-dose PERK inhibitors have been shown to restore suppressed glucose-stimulated insulin secretion (GSIS) and improve calcium signaling *in vitro*. Furthermore, in high-fat diet-induced diabetic mice, they enhanced GSIS, lowered blood glucose, and improved glucose tolerance (86). These findings suggest that carefully titrated PERK inhibition may improve β-cell function under selected metabolic-stress conditions. Whether such benefits can be achieved without compromising the essential adaptive functions of PERK remains uncertain.

The need to carefully control the extent and duration of PERK inhibition is underscored by the pancreatic toxicity observed after systemic GSK2606414 treatment. Sustained treatment caused pancreatic tissue loss and exocrine injury, together with hyperglycemia, reduced insulin secretion, and impaired glucose tolerance in mouse models (87, 88). Moreover, GSK2606414 also inhibits KIT and RIPK1, indicating that some pharmacological effects observed in relevant cellular contexts may not be attributable solely to PERK inhibition (89, 90). Together, these findings suggest that the therapeutic window for pharmacological PERK inhibition may be narrow and support the development of more selective, dose- and time-controlled approaches with careful pancreatic safety monitoring.

### 5.2 Modulating downstream translational control: ISRIB and eIF2B activation

Compared to directly targeting upstream kinases, modulating the downstream translation initiation complex of the ISR is considered another viable strategy. ISRIB (Integrated Stress Response Inhibitor) is a small-molecule compound that reverses the translational inhibition caused by eIF2α phosphorylation. It does so by stabilizing the guanine nucleotide exchange factor activity of eIF2B (eukaryotic initiation factor 2B), thereby restoring protein synthesis (91, 92). The mechanism of action of ISRIB is unique. It does not directly inhibit eIF2α kinases but acts downstream on eIF2B, reversing the inhibition of eIF2B nucleotide exchange activity imposed by phosphorylated eIF2α, thus restoring translational activity (92). In a mouse model, a 4-week ISRIB intervention attenuated ISR signaling, preserved β-cell mass, improved glucose tolerance, and restored the expression of β-cell maturity markers, including UCN3 and GLUT2 (93).

Nevertheless, evidence for ISRIB in diabetes remains confined to preclinical models, and its efficacy and safety in patients are unknown. The principal concern is whether eIF2B activation can reverse maladaptive translational repression without removing the protective translational brake required during unresolved ER stress. Premature restoration of protein synthesis may increase proinsulin folding demand before ER capacity has recovered, thereby worsening β-cell proteostatic stress. Future studies should define the disease stage and stress-resolution state in which ISRIB produces sustained β-cell benefit, particularly in human islets and clinically relevant models.

### 5.3 Chemical chaperones: alleviating upstream folding load

Chemical chaperones indirectly modulate the ISR by stabilizing protein conformation and enhancing the capacity of the endoplasmic reticulum to handle misfolded proteins (94). TUDCA (tauroursodeoxycholic acid) and 4-PBA (4-phenylbutyric acid) are representative agents in this category (95). Foundational research has confirmed that TUDCA can suppress the activation of ER stress-related signaling (e.g., decreased p-PERK levels) in the liver and adipose tissue of diabetic mice, improving glucose tolerance and insulin sensitivity. However, these systemic effects do not establish direct correction of ISR dysregulation in pancreatic β-cells.

Compared with their systemic metabolic effects, β-cell-focused evidence for chemical chaperones remains largely preclinical. TUDCA reduced ER-stress-associated β-cell apoptosis and preserved insulin secretion through an ATF6-dependent adaptive UPR response (96). Meanwhile, 4-PBA attenuated high-glucose-induced CHOP expression and Caspase-3 activation in insulin-secreting cells, and reduced hyperglycemia and pancreatic amyloid deposition in diabetic mice (97, 98). However, these agents alleviate the upstream burden of ER protein folding rather than directly targeting the core ISR mechanism. Furthermore, although cell-based studies support the effects of TUDCA and 4-PBA on insulin-secreting cells under stress, animal studies have employed systemic administration. Consequently, the observed improvements in pancreatic function cannot be attributed solely to direct protection of β-cells, as metabolic changes in other tissues may also have played a role. These findings position chemical chaperones as an indirect means of modulating endoplasmic reticulum stress within therapies targeting ISR. However, their specific efficacy regarding β-cells in human diabetes remains to be established.

### 5.4 Repurposing of existing drugs: the stress-modulating effects of GLP-1 receptor agonists

Beyond novel molecules, some medications already used in diabetes management have also been shown to influence endoplasmic reticulum stress and ISR-related processes, thereby promoting β-cell adaptation and survival. In animal models, GLP-1 receptor agonists (such as Exendin-4) have been found to reduce biochemical markers of islet ER stress and decrease β-cell death (99). GLP-1R signaling enhances the translation of ATF4 under ER stress conditions and accelerates the recovery of cells from eIF2α-mediated translational inhibition. This process is dependent on the cAMP/PKA pathway and is accompanied by the restoration of protein synthesis capacity (99).

These findings suggest that GLP-1 receptor activation may support adaptive recovery from ER stress rather than simply suppress ISR signaling. However, whether ISR modulation contributes substantially to the β-cell benefits of clinically used GLP-1 receptor agonists in humans remains uncertain.

### 5.5 Biomarker-guided evaluation of ISR-directed therapies

A major obstacle to evaluating ISR-directed therapies is the absence of a validated circulating biomarker that directly reflects ISR activity in pancreatic β-cells. Because transient ISR activation supports stress adaptation whereas sustained activation may promote β-cell dysfunction, therapeutic monitoring should determine whether excessive stress is reduced without compromising β-cell function. Circulating GDF15 and FGF21 are candidate markers for systemic stress responses associated with the ISR. However, studies involving skeletal muscle indicate that these markers are not tissue-specific, which limits their utility as markers for assessing target engagement specific to β-cells (100–102). β-cell outcomes should therefore be evaluated separately. Stimulated C-peptide reflects residual secretory capacity. The proinsulin-to-C-peptide ratio provides an indirect measure of impaired proinsulin processing and β-cell stress. Circulating unmethylated INS DNA remains an exploratory marker of β-cell death (103–106). No single marker can currently distinguish adaptive from maladaptive ISR activation. Future prospective studies should longitudinally assess circulating GDF15 and FGF21 together with stimulated C-peptide and markers of β-cell stress or death. These studies should determine whether this combined approach can monitor ISR-related stress and β-cell functional reserve. Its value for evaluating responses to ISR-directed therapies should also be established.

In summary, ISR-directed strategies act at four related levels: upstream kinase activity, downstream translational control, ER protein-folding capacity, and the broader cellular stress environment. Their shared aim is to attenuate maladaptive stress while preserving adaptive ISR signaling. Most supporting evidence remains preclinical, and the optimal dose, timing, tissue specificity, long-term efficacy, and safety of these interventions remain unresolved. Biomarker-guided studies will therefore be essential for defining therapeutic windows, monitoring target engagement, and identifying patients most likely to benefit.

## 6 Conclusion and future outlook

The integrated stress response (ISR) has emerged as a central regulator of pancreatic β-cell fate in diabetes, linking endoplasmic reticulum stress, metabolic overload, oxidative stress and inflammatory cues to translational reprogramming and cell-state change. In β-cells, which are uniquely challenged by sustained insulin biosynthesis and secretory demand, ISR signaling is inherently double-edged. When transient and properly resolved, it supports adaptation by reducing protein-folding burden and preserving cellular homeostasis. By contrast, when prolonged or dysregulated, it shifts from a protective program to a driver of proteostasis collapse, β-cell dedifferentiation, impaired insulin production and apoptosis, thereby accelerating the progressive loss of functional β-cell mass.

A major challenge is that ISR dysregulation is shared across multiple forms of diabetes, yet arises from distinct pathogenic contexts. In monogenic diabetes, human genetics demonstrates the essential role of intact ISR signaling in β-cell homeostasis. In type 1 diabetes, ISR appears to operate not only within β-cells but also within the inflammatory microenvironment. In type 2 diabetes, chronic nutrient excess and secretory overload promote a persistent ISR state that increasingly becomes maladaptive rather than compensatory. These observations argue against uniform ISR inhibition and instead support context-dependent, stage-specific modulation.

Future studies should define the thresholds, kinetics and reversibility of the transition from adaptive to maladaptive ISR, clarify the context-specific roles of PERK, GCN2, PKR and HRI, and resolve how ISR interfaces with mitochondrial dysfunction, inflammation and β-cell identity loss. Such advances may establish ISR not only as a mechanistic framework for β-cell failure, but also as a tractable axis for precision therapy in diabetes.
